# BIM-23A760 influences key functional endpoints in pituitary adenomas and normal pituitaries: molecular mechanisms underlying the differential response in adenomas

**DOI:** 10.1038/srep42002

**Published:** 2017-02-09

**Authors:** Alejandro Ibáñez-Costa, Laura M. López-Sánchez, Manuel D. Gahete, Esther Rivero-Cortés, Mari C. Vázquez-Borrego, María A. Gálvez, Andrés de la Riva, Eva Venegas-Moreno, Luis Jiménez-Reina, Alberto Moreno-Carazo, Francisco J. Tinahones, Silvia Maraver-Selfa, Miguel A. Japón, Juan A. García-Arnés, Alfonso Soto-Moreno, Susan M. Webb, Rhonda D. Kineman, Michael D. Culler, Justo P. Castaño, Raúl M. Luque

**Affiliations:** 1 Maimonides Institute of Biomedical Research of Cordoba (IMIBIC); 14004 Cordoba, Spain; 2 Department of Cell Biology, Physiology and Immunology, University of Cordoba; 14004 Cordoba, Spain; 3 Hospital Universitario Reina Sofía (HURS); 14004 Cordoba, Spain; 4 CIBER de la Fisiopatología de la Obesidad y Nutrición (CIBERobn); 14004 Cordoba, Spain; 5 Campus de Excelencia Internacional Agroalimentario (ceiA3); 14004 Cordoba, Spain; 6 Service of Endocrinology and Nutrition, IMIBIC, HURS, 14004 Cordoba, Spain; 7 Service of Neurosurgery, HURS, 14004 Cordoba, Spain; 8 Metabolism and Nutrition Unit, Hospital Universitario Virgen del Rocío, Instituto de Biomedicina de Sevilla (IBIS), 41013 Seville, Spain; 9 Department of Morphological Sciences, University of Cordoba, 14004 Cordoba, Spain; 10 Endocrinology and Nutrition Unit, Complejo Hospitalario de Jaén, 23007 Jaén, Spain; 11 Service of Endocrinology and Nutrition, Hospital Clínico Universitario Virgen de la Victoria, 29010 Málaga, Spain; 12 Department of Pathology, Hospital Universitario Virgen del Rocío, 41013 Seville, Spain; 13 Department of Endocrinology and Nutrition, Carlos Haya Hospital, 29010 Málaga, Spain; 14 Department of Endocrinology, Hospital Sant Pau, Centre for Biomedical Research on Rare Diseases (Centro de Investigación Biomédica en Red de Enfermedades Raras Unit 747) Autonomous University of Barcelona; 08035 Barcelona Spain; 15 Department of Medicine, University of Illinois at Chicago, Jesse Brown Veterans Affairs Medical Center, Research and Development Division, Chicago, Illinois, USA; 16 IPSEN Bioscience, Cambridge, 02142 Massachusetts, USA

## Abstract

Chimeric somatostatin/dopamine compounds such as BIM-23A760, an sst2/sst5/D_2_ receptors-agonist, have emerged as promising new approaches to treat pituitary adenomas. However, information on direct *in vitro* effects of BIM-23A760 in normal and tumoral pituitaries remains incomplete. The objective of this study was to analyze BIM-23A760 effects on functional parameters (Ca^2+^ signaling, hormone expression/secretion, cell viability and apoptosis) in pituitary adenomas (n = 74), and to compare with the responses of normal primate and human pituitaries (n = 3–5). Primate and human normal pituitaries exhibited similar sst2/sst5/D2 expression patterns, wherein BIM-23A760 inhibited the expression/secretion of several pituitary hormones (specially GH/PRL), which was accompanied by increased sst2/sst5/D2 expression in primates and decreased Ca^2+^ concentration in human cells. In tumoral pituitaries, BIM-23A760 also inhibited Ca^2+^ concentration, hormone secretion/expression and proliferation. However, BIM-23A760 elicited stimulatory effects in a subset of GHomas, ACTHomas and NFPAs in terms of Ca^2+^ signaling and/or hormone secretion, which was associated with the relative somatostatin/dopamine-receptors levels, especially sst5 and sst5TMD4. The chimeric sst2/sst5/D_2_ compound BIM-23A760 affects multiple, clinically relevant parameters on pituitary adenomas and may represent a valuable therapeutic tool. The relative ssts/D_2_ expression profile, particularly sst5 and/or sst5TMD4 levels, might represent useful molecular markers to predict the ultimate response of pituitary adenomas to BIM-23A760.

Pituitary adenomas represent one of the most common intracranial neoplasms. They are often accompanied by serious comorbidities, due to excessive hormonal secretion and/or compression of intracranial structures, such as amenorrhea, galactorrhea, growth abnormalities, hypopituitarism, cognitive and emotional disturbances and sexual dysfunctions^1^,^2^,^3^,^4^,^5^.

Somatostatin (SST) and dopamine (DA) are two well-known factors that regulate numerous, often overlapping, (patho)physiological functions^6^,^7^,^8^. Both, SST and DA bind to its own family of receptors (sst1–5 and D_1–5_, respectively), which exhibit a wide expression pattern in normal and tumoral tissues, including pituitary adenomas^7^,^9^. Activation of SST- and DA-receptors results in multiple, mostly inhibitory actions on endocrine and/or exocrine hormonal secretions and cellular proliferation^7^,^8^. Accordingly, these receptors serve as valuable targets for the pharmacological management of pituitary adenomas and other tumoral pathologies. Interestingly, pituitary adenomas often express, simultaneously, high levels of various ssts and Ds, showing expression profiles substantially altered compared with normal pituitaries or to cell types from which pituitary adenoma are originated^10^,^11^. Based on this and additional evidences, pharmaceutical companies have developed functional compounds selective for one or multiple sst-subtypes, with those selective for sst2 and sst5 being particularly useful (e.g., lanreotide, octreotide)^8^. Similarly, DA agonists selective for D_2_ (e.g., cabergoline), have been also generated and are efficiently used to treat some pituitary adenomas types, especially prolactin-secreting adenomas^12^.

Although ssts and Ds are highly present in pituitary adenomas, and the efficiency of the individual selective sst2/5 or D_2_ compounds have been proven in the treatment of pituitary adenomas, an appreciable subset of patients are poorly responsive or totally resistant to conventional therapy with SST- or DA-analogs^8^,^12^,^13^. Therefore, new approaches are already being tested or are currently under clinical investigation, such as the use of combined therapies (SST- plus DA-analogs), which have been shown to be more effective than individual compounds^8^.

Therefore, based on the well-known interaction between the SST- and DA-systems^14^ and on the ability of sst2 and sst5 to physically and functionally interact with D_2_ resulting in altered pharmacological or/and signaling properties^14^,^15^, an interesting new approach that is currently under basic and clinical investigation is the development and application of chimeric SST/DA compounds. As previously shown^16^, these drugs can retain the ability to interact with ssts and D_2_, and can display greater effects in reducing pituitary secretions than individual compounds. One of these promising chimeric SST/DA compounds is BIM-23A760, an agonist for sst2/sst5/D_2_ receptors used in clinical trials^17^,^18^,^19^. Specifically, the effect of BIM-23A760 has been tested in pituitary cell lines^20^ and in limited series of primary pituitary adenoma cell cultures^16^,^21^,^22^,^23^,^24^,^25^,^26^; however, the data collected to date on the *in vitr*o effect of BIM-23A760 in pituitary adenomas is still incomplete, with some apparently contradictory results, which renders the available evidence somewhat inconclusive. Moreover, previous studies have not been focused to specifically analyze and compare the distinct effects of BIM-23A760 on several functional parameters in parallel in the same samples and/or in a wide range of cell types from pituitary adenomas, nor the responses of these pituitary adenoma cells have been compared with those observed in normal pituitary cells *in vitro*. Therefore, the aim of this study was to analyze a set of relevant functional parameters (Ca^2+^ signaling, hormonal expression and secretion, cell viability and apoptosis), in response to BIM-23A760 in the main types of pituitary adenomas, and in human/primate normal pituitary cells. Although BIM-23A760 has been withdrawn from clinical development after discovering a dopaminergic metabolite that accumulates and interferes with the activity of the parent compound *in vivo*^19^,^27^, it is still considered a good prototype molecule and therefore, the results generated using primary pituitary cell cultures from pituitary adenomas and normal pituitaries may be really useful in predicting the response to members of this class of compounds (i.e. new generation of chimeric agonist for sst2/sst5/D_2_ receptors) that may be used for clinical purposes in the future.

## Results

### Expression profile of sst2, sst5 and D2 in human and baboon normal pituitaries

**Figure 1:**
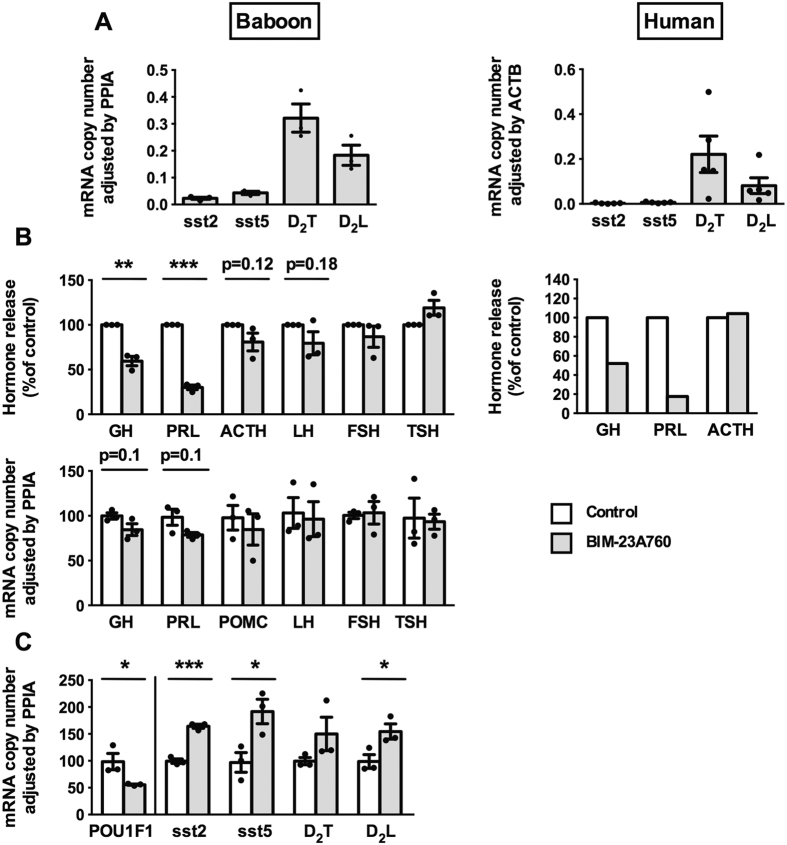
Expression profile and hormone release in the presence or absence of BIM-23A760 in normal pituitaries from baboons and humans. (**A**) Expression profile of sst2, sst5, D_2_ (Total and Long) receptors in whole pituitary of female baboons (n = 3) and human pituitary (n = 5). (**B**) Effect of BIM-23A760 (100 nM) after 4-h treatment on the secretion and mRNA expression of all pituitary hormones in baboon primary pituitary cell cultures (n = 3; top and bottom panel on the left, respectively), and 24-h treatment on GH, PRL and ACTH release in human primary pituitary cell culture (n = 1; right-panel). (**C**) Effect of BIM-23A760 on POU1F1, sst2, sst5, D_2_ (Total and Long) after 4-h treatment in baboon primary pituitary cell cultures (n = 3). Values in figure B/C are expressed as percent of vehicle-treated controls, set at 100% within experiment. Hormonal release was determined by commercial ELISA kits. mRNA expression levels were measured by qPCR, and mRNA copy numbers were adjusted by Cyclophilin A (PPIA) and beta-actin (ACTB) mRNA copy number expression in baboons and humans, respectively. Values represent the mean ± SEM (3–4 wells/treatment/experiment). Asterisks show significant differences between BIM-23A760 and vehicle-treated controls in figure B and C (*p < 0.05, **p < 0.01; ***p < 0.001). In cases where only one experiment (n = 1) could be performed, no error bars are presented and no significance tests were performed.

We found that sst5, sst2 and D_2_ were highly expressed in both baboon and human pituitary (Fig. 1A). Notably, the expression profile of sst2/sst5/D_2_, the target receptors for BIM-23A760, was virtually identical in both species (Fig. 1A). Specifically, pituitary cultures of baboons (n = 3) and humans (n = 5) cells expressed high levels of sst5, sst2 and D_2_, with relative order of D_2_T > D_2_L > sst5 > sst2. Notably, we found that cultures of baboon normal pituitaries maintain the same expression profile after dispersion and culture as whole normal pituitary tissues from baboons (Supplemental Table 1) which, together with the results presented in Fig. 1A, suggest that the baboon normal pituitary cultures might represent an appropriate model to study how BIM-23A760 modulate human pituitary cell function.

Incubation of cultured baboon pituitary cells with BIM-23A760 revealed clear inhibitory effects on GH and PRL release and a tendency to inhibit their expression levels (Fig. 1B, left-panels). Conversely, expression and/or release of proopiomelanocortin (ACTH and POMC, the ACTH-precursor, respectively), LH, FSH or TSH were not significantly altered in response to BIM-23A760 (Fig. 1B, left-panels). Because of the limited available amount of human normal pituitary samples, we could not study in depth the effects of BIM-23A760 on human normal pituitaries; however, we obtained a limited number of cells derived from a dispersed human normal pituitary preparation to study the effect of BIM-23A760 on the secretion of some, selected, hormones. Of note, as observed in baboons, we found that BIM-23A760 treatment seemed to inhibit GH and PRL, but not ACTH release in this culture of human normal pituitary cells (Fig. 1B, right-panel). Interestingly, we also observed that BIM-23A760 treatment evoked an up-regulation of sst2, sst5 and D_2_L expression, and a down-regulation of POU1F1 mRNA levels in baboons normal pituitary cell cultures (Fig. 1C).

### Direct effects of BIM-23A760 on [Ca^2+^]_i_ levels in human pituitary adenomas and normal pituitaries

It should be mentioned that free cytosolic calcium ([Ca^2+^]_i_) acts as a second messenger well-known for being directly involved and required in hormone secretory vesicle release^28^. The measurement of [Ca^2+^]_i_ provides useful information about several parameters including: (1) the percentage of maximum response (PMR), which indicates the maximal response (positive or negative) achieved in [Ca^2+^]_i_ in response to a specific treatment; (2) the proportion of responsive cells (PRC), which indicates the percentage of cells that elicit a positive or negative response in terms of [Ca^2+^]_i_ levels in response to an specific treatment; and (3) the time to maximal response of sensitive cells to the specific compound, which indicates the time when the maximal response (positive or negative) is achieved in terms of [Ca^2+^]_i_ levels in response to the specific treatment^29^,^30^. BIM-23A760 treatment inhibited [Ca^2+^]_i_ levels (Table 2) in cell cultures of all normal pituitaries tested (n = 4; PMR: 77%, PRC: 42%), in 65% of GHomas (n = 13/20; PMR: 72%, PRC: 64%), in 80% of mixed GH/PRLomas (n = 4/5; PMR: 79%, PRC: 42%), in 67% of PRLomas (n = 4/6; PMR: 69%, PRC: 30%), in 30% of ACTHomas (n = 3/10; PMR: 73%, PRC: 24%), in 25% of NFPA (n = 4/16; PMR: 81%, PRC: 44%), in the available FSHoma (PMR: 65%, PRC: 66%), and in one of the two TSHomas included in the study (PMR: 53%, PRC: 65%). BIM-23A760 did not alter [Ca^2+^]_i_ levels in 5%, 20%, 33%, 40%, 63% and 50% of the GHomas, mixed GH/PRLomas, PRLomas, ACTHomas, NFPAs and TSHomas analyzed, respectively (Table 2). Interestingly, we also found that BIM-23A760 increased [Ca^2+^]_i_ levels in 30% of GHomas and ACTHomas (PMR: 212 and 223%; PRC: 69 and 35%, respectively), and in 13% of the NFPAs analyzed (PMR: 170%, PRC: 19%). Representative profiles depicting changes in [Ca^2+^]_i_ levels in normal and tumoral pituitary cell cultures in response to BIM-23A760 are presented in Supplemental Figure 1.

### Hormonal expression/release

**Figure 2:**
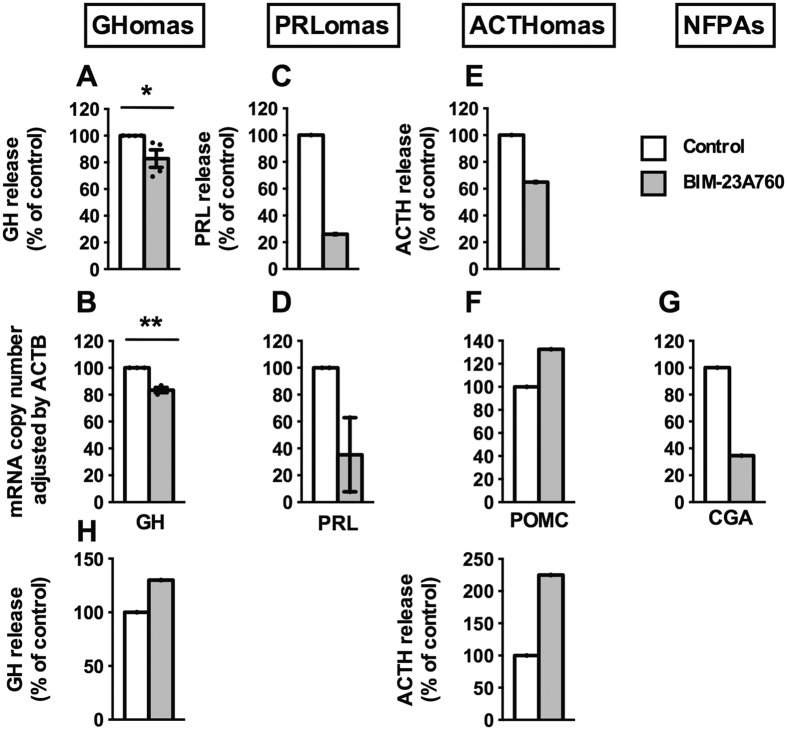
Effect of BIM-23A760 (100 nM; 24 h) on pituitary hormone release and/or expression in primary human pituitary cell cultures from GHomas (**A** and **B**; n = 3–4), PRLomas (**C** and **D**; n = 1–2), ACTHomas (**E** and **F**; n = 1) and NFPAs (**G**; alpha-subunit (CGA); n = 1). (**H**) Stimulatory effect of BIM-23A760 on GH and ACTH release in GHomas and ACTHomas (n = 1). Values are expressed as percent of vehicle-treated controls, set at 100% within experiment. Hormonal release was determined by commercial ELISA kits, and mRNA expression levels were measured by qPCR. mRNA copy numbers were adjusted by ACTB mRNA copy number. Values represent the mean ± SEM. Asterisks show significant differences between BIM-23A760 and vehicle-treated controls (*p < 0.05, **p < 0.01). In cases where only one experiment (n = 1) could be performed, no error bars are presented and no significance tests were performed.

Incubation with BIM-23A760 (24 h) inhibited GH release (Fig. 2A) and expression (Fig. 2B) in GHomas. It also seemed to decrease PRL release/expression in PRLomas (Fig. 2C–D) and ACTH release in one ACTHoma (Fig. 2E) but not POMC expression (Fig. 2F). BIM-23A760 also seemed to reduce CGA expression in one NFPA (Fig. 2G); whereas it increased hormonal levels in one GHoma and one ACTHoma (Fig. 2H).

### Cell viability and apoptotic rate

**Figure 3:**
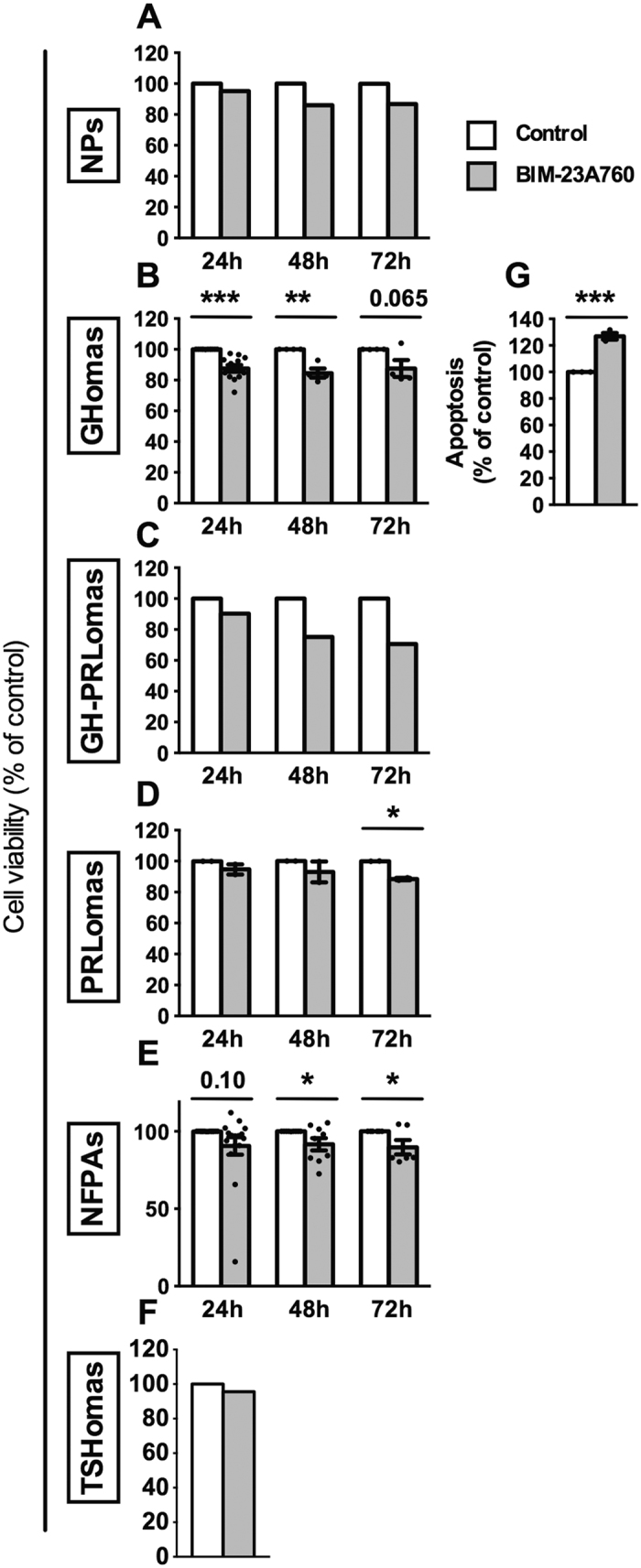
(**A**) Effect of BIM-23A760 (100 nM) on cell viability (24–72-h treatment) in human normal pituitaries (NP; n = 1), GHomas (n = 11), mixed GH/PRLomas (n = 1), PRLomas (n = 2), NFPAs (n = 18) and TSHomas (n = 1). Effect of BIM-23A760 on apoptosis in GHomas (n = 3). Values represent the mean ± SEM. Asterisks show significant differences between BIM-23A760 and vehicle-treated controls (*p < 0.05, ***p < 0.001). In cases where only one experiment (n = 1) could be performed, no error bars are presented and no significance tests were performed.

Direct effects of BIM-23A760 on cell viability and apoptotic rate were also tested in human normal pituitaries and pituitary adenomas cultures. BIM-23A760 seemed to moderately inhibit cell viability at different incubation-times in one cultured normal pituitary (Fig. 3A). Similarly, BIM-23A760 also inhibited cell viability in GHomas (Fig. 3B), one mixed GH/PRLoma (Fig. 3C) and NFPAs (Fig. 3E). Additionally, BIM-23A760 down-regulated cell viability in PRLomas at 72 h (Fig. 3D), but had no effect in the TSHoma analyzed (Fig. 3F). Moreover, BIM-23A760 increased apoptotic rate in GHomas, as observed using Annexin-V-FITC/propidium iodide staining assay (Fig. 3G).

### Molecular mechanisms underlying the differential, inhibitory/stimulatory, response to BIM-23A760 in human GHomas and ACTHomas

**Figure 4:**
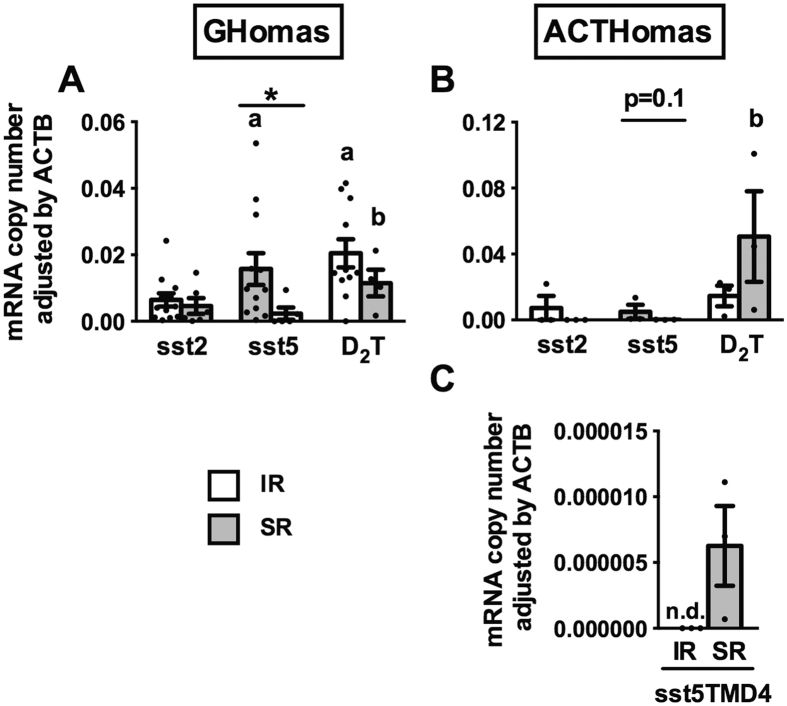
Differential expression profile of sst2, sst5 and D_2_T according to the inhibitory and stimulatory responses observed in terms of [Ca^2+^]_i_ kinetics (Table 2) in GHomas (**A**; n = 12, and n = 6, inhibited- and stimulated- adenomas, respectively) and ACTHomas (**B**); n = 3 and n = 3, inhibited- and stimulated- adenomas populations. Inhibitory and stimulatory responders were indicated as IR (white bars) and SR (black bars), respectively. (**C**) Expression profile of sst5TMD4 in the inhibited population (n = 3) and stimulated population (n = 3) of ACTHomas. Values represent the mean ± SEM. Asterisks show significant differences between the same receptor-subtype in inhibited- and stimulated- adenoma populations of GHomas and ACTHomas (*p < 0.05). “a” indicates a statistical difference in the expression levels of sst5 or D_2_ as compared with sst2 expression in the inhibited population. “b” indicates a statistical difference in the expression levels between of sst5 compared with D_2_ expression in the stimulated population.

The results previously presented suggest that two populations may exist in pituitary adenomas (at least within GHomas and ACTHomas) that respond differentially, even oppositely, to BIM-23A760 (hence forth referred to as “inhibited pituitary adenoma” and “stimulated pituitary adenoma”). Analysis and comparison of the expression levels profile of D_2_ and sst2/sst5-subtypes in human GHomas and ACTHomas revealed that D_2_ and sst5 expression levels were significantly higher than sst2 in inhibited GHomas (Fig. 4A); whereas, sst5 levels were lower than those of D_2_ in stimulated GHomas. When comparing the expression levels across GHomas, the only difference found was a significantly lower sst5 expression in stimulated GHomas vs. inhibited GHomas (Fig. 4A). In ACTHomas, there were no differences between the average expression levels of sst2, sst5 and D_2_ in inhibited ACTHomas (Fig. 4B). In contrast, D_2_ expression levels were significantly higher than those of sst2 and sst5 in stimulated ACTHomas. Interestingly, when expression levels were compared across ACTHomas, we found a similar situation to that previously observed in GHomas, in that sst5 levels were lower in stimulated vs. inhibited ACTHomas (Fig. 4B).

Finally, truncated sst5TMD4 expression levels did not change between both GHomas populations (mean of 187 copies/0.05 μg of total RNA); however, the stimulated population of ACTHomas expressed higher mRNA levels of sst5TMD4 (mean of 96 copies/0.05 μg of total RNA), while no expression was detectable in the inhibited population of ACTHomas (0 copies/0.05 μg of total RNA; Fig. 4C).

## Discussion

The results of this study indicated that, consistent with previous reports^11^,^31^, sst5, sst2 and D_2_ were highly expressed in both baboon and human normal pituitaries, showing a similar expression pattern (i.e. D_2_T > D_2_L > sst5 > sst2). These results, coupled with our data showing that baboon pituitary cultures maintain the same expression profile after dispersion and culture as whole-pituitaries, and with previous reports aimed to analyze pituitary physiology^32^,^33^, strongly suggest that baboon normal pituitaries might be an appropriate model to study human pituitary function. Thus, *in vitro* treatment of cultured baboon pituitary cells with BIM-23A760 demonstrated a decrease of GH and PRL expression/release, which was comparable to that shown in human pituitary. To the best of our knowledge, this is the first study showing, the direct effect of *in vitro* BIM-23A760 on the expression and/or secretion of all major pituitary hormones in primates and/or humans normal pituitaries. As such, our results extend and reinforce previous data, which indicated that s.c. administration of BIM-23A760 suppressed circulating PRL secretion in healthy male volunteers^17^, and GH, IGF1 and PRL secretion in normal cynomolgus monkeys *in vivo*^34^. Furthermore, the present work demonstrates that BIM-23A760 effects on normal pituitary are not just confined to regulate the release or expression of pituitary hormones, but also include an up-regulation of sst2, sst5 and D_2_L expression. These findings suggest that, even though mRNA may not always necessarily reflect the precise, final protein levels of functional receptors available at the cell membrane (a technically challenging assay that we could not perform given the limited tissue availability), the changes observed herein likely represent a relevant up-regulation in the expression of these receptors, which would reflect an additional regulatory mechanism, that is typically observed in GPCRs^33^, on pituitary cells^35^, and enables to finely tune the response of pituitary cells to their ligands. Moreover, our data invite to speculate that the effects of BIM-23A760 on somatotropes/lactotropes might be mediated by the inhibition of POU1F1 mRNA production. Altogether, these novel results support the notion that the use of chimeric molecules as BIM-23A760 could provide a new, valuable therapeutic tool for medical treatment of different pituitary adenoma types, and thereby invite further exploration of the underlying molecular mechanisms of its effects.

The central aim of this study was to systematically analyze the effect of BIM-23A760 on essential functional parameters in all major human pituitary adenoma cell types. Because of the limited cells available after surgical resection and dispersion of pituitary adenomas, we decided to test the effect of BIM-23A760 on [Ca^2+^]_i_ kinetics assay, a sensitive assay that requires a small number of cells compared to other assays and provides useful information about several parameters (PRC, PMR,…). Our data indicate that BIM-23A760 inhibited [Ca^2+^]_i_ levels in normal and tumoral pituitary cells; but increased [Ca^2+^]_i_ levels in a population of GHomas, ACTHomas and NFPAs, demonstrating that BIM-23A760 directly acts on human pituitary adenomas and normal pituitary cells by activating [Ca^2+^]_i_ levels. In this regard, the only previous study exploring activation of signaling pathways in response to BIM-23A760 indicated that the antiproliferative effect of BIM-23A760 involved phosphorylation of ERK1/2 and p38 MAPK pathways, and that this antiproliferative effect was mainly exerted through the activation of D_2_ in NFPAs^23^. However, our study is the first to characterize, in a wide range of pituitary adenomas and in normal pituitaries, a key element of the signaling pathway activated by BIM-23A760 directly linked to hormone release, i.e. [Ca^2+^]_i_^28^. When remaining cells were available from normal pituitaries and pituitary adenomas cultures, we decided to additionally measured hormonal secretion and/or expression, cell viability and apoptosis in response to BIM-23A760 but, unfortunately, the limited amount of cells available precluded the desired in-depth study on the possible mechanisms associated with these changes in [Ca^2+^]_i_ on human pituitary adenoma and normal pituitary cells in response to BIM-23A760. Nevertheless, the question still arises, why some pituitary adenomas exhibit a differential, even opposite, response to BIM-23A760? As will be discussed further below, a different receptor expression profile, and the possible interactions between receptors in the various pituitary adenoma types, might provide at least a partial explanation for the effects of BIM-23A760.

We found that treatment with BIM-23A760 inhibited hormone secretion and/or expression in GHomas; and in individual observations in PRLomas, ACTHomas and NFPAs, which is in line with previous reports showing that BIM-23A760 potently decreased hormone release in cultures from GHomas and the GH_3_ cell line^16^,^20^,^26^,^36^, PRLomas and the MMQ cell line^20^,^22^, mixed GH/PRLomas^16^,^26^ and ACTHomas^37^, and it also agrees with a Phase-II clinical study in acromegalic patients showing that BIM-23A760 inhibits basal circulating GH levels^18^,^19^. In favor of the idea of using chimeric SST/DA compounds to reduce hormonal expression/secretion from various pituitary adenoma types is the report of *Saveanu* and coauthors demonstrating that the potency of another chimeric compound (BIM-23A387) in suppressing GH secretion was 100-times higher than individual sst2 or D_2_ analogs^38^. However, it should be noted that, in line with both the stimulatory and inhibitory effects of BIM-23A760 observed on [Ca^2+^]_i_ levels, we also observed that BIM-23A760 increased GH and ACTH release in individual cases of GHomas and ACTHomas, which suggest that there are different subpopulations of adenomas. We could not corroborate whether NFPAs also have a differential response to BIM-23A760 as these tumors respond poorly in terms of hormone secretion.

Hence, and despite exercising due caution, our results reinforce the notion that at least two populations may exist in GHomas and ACTHomas that respond differentially, even oppositely, to BIM-23A760. Nevertheless, this stimulatory effect of BIM-23A760 should not necessarily be surprising, based on previous studies which have revealed that SST, DA and/or their analogs can directly stimulate pituitary hormone secretion in primates and other species^31^,^39^,^40^, and in a select population of human pituitary adenomas^40^,^41^,^42^. Furthermore, it is well-known that different SST-agonists can elicit different effects in the same pituitary adenoma cell-type, known as “biased agonism”^43^, which might be directly depending on the agonist-receptor and receptor-receptor interactions, on the active receptor-conformations, etc. Therefore, and although we could not perform a systematical analysis of the relative contribution of SST and DA receptors to this differential response due to a limitation in the availability of cells from the same pituitary adenoma, comparison of the receptor expression profiles between the pituitary adenoma inhibited-population and stimulated-population in response to BIM-23A760 might be important for determining the molecular mechanism involved in the opposite responses to this and other compounds (see below).

We also observed that BIM-23A760 treatment decreased cell viability in GHomas, NFPAs and PRLomas at different incubation-times and stimulates apoptosis in GHomas. These results are, in part, consistent with previous data showing that BIM-23A760 decreased cell viability/proliferation in NFPAs^21^,^23^,^44^ and TSHomas^25^, and increased apoptosis in NFPAs^44^. These results, therefore, suggest that in addition to suppressing [Ca^2+^]_i_ levels and hormonal hypersecretion, BIM-23A760 may be able to decrease cell viability and increase apoptotic rate in pituitary adenomas. Taken together, our results provide relevant information from a mechanistic/therapeutic perspective, since demonstrate that BIM-23A760 can alter (decrease) key cellular parameters and also influences clinically relevant endpoints in a significant subset of pituitary adenomas. Once again, these findings invite further exploration of the potential value of SST-DA chimeric compounds as therapeutic approaches for patients with pituitary adenomas.

In order to identify molecular determinants that could explain the differential, inhibitory/stimulatory, responses to BIM-23A760, expression levels of sst2/sst5/D_2_ were measured in GHomas and ACTHomas and the results were compared between “inhibited pituitary adenomas” and “stimulated pituitary adenomas”, based on the data of [Ca^2+^]_i_ kinetics. Interestingly, when results from both GHomas and ACTHomas are viewed together, we observed two common, distinctive molecular signatures, namely, a lower sst5 expression in stimulated vs. inhibited pituitary adenomas, and a higher D_2_ expression level as compared with sst5 expression in stimulated pituitary adenomas. Therefore, these findings might suggest that the relative sst5 expression levels (i.e. lower sst5 levels in stimulated vs. inhibited pituitary adenomas as well as, lower sst5 as compared with D_2_ levels in the stimulated pituitary adenomas) might represent a potential molecular signature contributing to the differential response to BIM-23A760.

These results, coupled to our previous demonstration that the truncated sst5TMD4 receptor is overexpressed in pituitary adenomas and plays relevant roles in GHomas^29^,^45^,^46^ and in other endocrine-related tumors^47^,^48^, prompted us to analyze its expression in the two pituitary adenoma populations. Notably, while sst5TMD4 expression levels did not change between both GHomas populations, the stimulated-population of ACTHomas expressed appreciable levels of sst5TMD4, which were not detectable in the inhibited-population of ACTHomas. Although full-length sst5 mRNA is the dominant sst5 gene transcript in the stimulated population of ACTHomas, with absolute levels much greater than those of sst5TMD4 (27-fold), our results unveil that sst5TMD4 is selectively expressed in the stimulated population of ACTHomas, thus suggesting a potential involvement of sst5TMD4 in the unique stimulatory response of some ACTHomas to BIM-23A760.

Nevertheless, when viewed together, our results strongly suggest that sst5 expression in GHomas and ACTHomas, and sst5TMD4 expression in ACTHomas might represent useful molecular markers to predict the ultimate response of these pituitary adenoma types to BIM-23A760. This idea is supported by a study showing that presence of sst5TMD4 is linked to a reduced *in vivo* hormonal response to SST-analogue treatment in acromegalic patients^45^. Further support that the low sst5 expression level in the stimulated-population of GHomas and ACTHomas may serve as a potential signature to distinguish between the differential response of pituitary adenomas is provided by a previous study in patients with TSHomas, which showed that tumors displaying a stimulatory response to octreotide expressed relatively low levels of sst5 in comparison with sst2, while tumors with higher sst2 than sst5 expression obtained a beneficial response to octreotide^25^. Nevertheless, it should not be discounted that other factors could also contribute to the differential, inhibitory/stimulatory response of GHomas and ACTHomas to BIM-23A760. One of these factors could be the precise number of receptors and/or their specific proportion available on the tumor cells. In this scenario, it seems conceivable that a specific expression profile (such as high D_2_ vs. sst5 expression levels in stimulated-pituitary adenomas compared to similar D_2_/sst5 expression levels in inhibited-pituitary adenomas) could be a key molecular determinant for the response to BIM-23A760, as it could dictate the possible interactions between receptor subtypes (homo and/or heterodimerization). Obviously, further, specifically directed, studies will be required to formally prove this notion with regard to the effect of BIM-23A760 in pituitary adenoma cells. However, this concept is supported by several studies demonstrating that the therapeutic response of pituitary adenomas to different drugs is directly dependent on the relative expression pattern of both SST and DA receptors^10^,^49^. The existence of heterodimers between SST/DA receptors (e.g. sst5 and D_2_) that result in changes in the functional, pharmacological and signaling properties of the receptors is well established^14^. In particular, this concept is supported by results demonstrating that only the amount of dimers between sst5 and D_2_, and not between sst2 with D_2_, was directly and positively correlated with an enhanced antiproliferative effect of BIM-23A760 in prostate and lung cancer cell lines^50^.

Altogether, the results of the present study provide convincing evidence that chimeric compounds for the sst2/sst5/D_2_ system might represent valuable tools for the design and development of new therapeutic drugs for the management of certain pituitary adenomas and its associated comorbidities in the near future.

## Materials and Methods

### Reagents

All reagents used in this study were purchased from Sigma-Aldrich (St. Louis, MO, USA) unless otherwise specified. BIM-23A760 was kindly provided by IPSEN Bioscience (Cambridge, MA, USA) and prepared as previously described^26^; specifically, dry powder was dissolved in 0.01 N acetic acid containing 0.1% bovine serum albumin, aliquoted and stored at −20 °C. Fresh working solutions were prepared for each experiment from a new aliquot.

### Patients and animals tissue collection

Human pituitary specimens were obtained during transsphenoidal surgery (from 2008 to 2014) from a total of 74 patients [22 somatotropinomas (GHomas), 5 mixed GH/PRLomas, 26 nonfunctioning pituitary adenoma (NFPAs), 6 PRL-secreting adenomas (PRLomas), 11 corticotropinomas (ACTHomas), 1 FSH-secreting gonadotropinoma (FSHoma) and 3 thyrotropinomas]. Moreover, 5 samples corresponding to normal pituitary tissue were used, which were obtained from patients who underwent surgical removal of a pituitary adenoma, and the tissue piece obtained by our laboratory was confirmed as normal pituitary tissue. Specifically, both human normal and tumoral pituitary tissue pieces were confirmed by 3 separate methods: examination by an expert anatomopathologist, molecular screening by quantitative real time PCR of the main pituitary hormonal products and membrane receptors, and analysis of the hormonal phenotype using single-cell secretion by a cell-blotting assay, as previously described^30^. Before surgery, all patients with a GHoma or a PRLoma were treated with somatostatin and dopamine analogs, respectively. Available patients’ demographic data are summarized in Table 1. Due to the extensive time-range required for collection of samples, histological and genetic information of the patients and samples obtained were limited, and therefore additional information about immunohistochemistry, granulation pattern of the tumors, presence of AIP or GNAS mutations, or other novel factors that have been demonstrated to affect the response to pharmacological treatment could not be provided^8^. Informed consent from each patient was obtained. Primate (Olive Baboon, *Papio anubis*; n = 3, 9–10 yr of age) pituitaries were obtained from randomly cyclic control females within 15 min after sodium pentobarbital overdose as previously reported^51^. It should be mentioned that the baboons used represent control animals from a breeding colony, all under Institutional Animal Care and Use Committee approved studies conducted by other University of Illinois at Chicago investigators. All the methods were carried out in accordance with the approved guidelines of the University of Córdoba/IMIBIC and Hospital Ethics Committees were obtained. All experimental protocols were approved by University of Córdoba, IMIBIC and University of Illinois at Chicago institutional committees.

### Primary pituitary cell culture

All human pieces (normal and tumoral pituitaries) were immediately collected after surgery, placed in sterile cold (4 °C) medium (S-MEM, Gibco, Madrid, Spain) supplemented with 0.1% BSA, 0.01% L-glutamine, 1% antibiotic-antimycotic solution, and 2.5% HEPES; then were rapidly moved to our laboratory on ice within 1–3 hours, where they were dispersed into single cells for culture by enzymatic and mechanical disruption following the methods and reagents previously reported^30^,^52^,^53^. Primate anterior pituitaries were cut into small pieces, and one-two fragments were rapidly frozen in liquid nitrogen and stored at −80 °C until extraction for total RNA, while the remaining pieces were placed in sterile cold α-Minimum essential medium (α-MEM) (Invitrogen, Grand Island, NY, USA) supplemented with 0.15% BSA, 6 mM HEPES, and 10 IU/ml penicillin and 10 μg/ml streptomycin (Invitrogen); and dispersed into single cells for culture following the methods/reagents previously reported^33^,^51^,^54^,^55^. Both human and primate pituitary samples were minced into 1–2 mm^3^ pieces under sterile conditions. Some pieces were stored for posterior RNA isolation at −80 °C and the remaining tissue was washed and incubated in 30 ml S-MEM medium complemented with 0.3% trypsin (Beckson, Dickinson and Company, Sparks, MD, USA) in a spinner flask (Bellco Glass, Vineland, NJ, USA) for 2 h at 37 °C under gentle shaking and then, incubation was continued for 5 min in presence of 1 mg of DNAse I (Roche, Mannheim, Germany). Dispersed cells were decanted by centrifugation and then, by repeated aspiration into a smooth tipped glass Pasteur pipette. Finally, human cell suspension was washed in 4.5 g/L glucose containing DMEM medium (Gibco, Madrid, Spain) complemented with 0.1% BSA, 0.01% L-glutamine, 1% antibiotic-antimycotic solution, and 2.5% HEPES, and baboon cell suspension was washed in supplemented α-MEM as above described but containing 10% horse serum (Invitrogen). To avoid fibroblast contamination, suspensions of dispersed human and baboon pituitary cells were filtered through a nylon gauze of 130 μm-mesh, and in both DMEM and α-MEM, D-Valine-was replaced for L-valine to selectively inhibit fibroblast proliferation/overgrowth as previously reported^56^. In addition, visual inspection of primary cell cultures at the time of experimental assays showed no sign of cells displaying the typical fibroblast-like morphology. All the individual pituitary cultures showed cell viability higher than 95%, as determined by the trypan blue dye exclusion method (American Type Culture Collection, Manassas, VA).

### Measure of pituitary hormones

Pituitary cell cultures (100,000–200,000 cell/well, n = 3–4 wells/treatment) were incubated with media alone (controls) or media containing BIM-23A760 (100 nM) for 4 h (primate normal pituitary cells) or 24 h (human normal pituitary and pituitary adenoma cells). At the end of the incubation with the corresponding treatment, media were recovered for hormone analysis and, whenever possible, cells were recovered for RNA analysis (see below). As previously reported elsewhere^51^, hormone concentrations were measured in the culture media derived from human samples according to the pituitary adenoma type and, when possible, all pituitary hormones concentrations were measured in the human and baboon normal pituitary samples using human/primate commercial ELISA kits [GH, LH, FSH, PRL, ACTH and TSH (reference numbers: EIA-3552, EIA-1289, EIA-1288, EIA-1291, EIA-3647 and EIA-1790, respectively; DRG, Mountainside, NJ, USA)] following the manufacturer’s instructions. All the information regarding specificity, detectability, and reproducibility for each of the assays can be accessed at the website of the company.

### RNA isolation, reverse transcription, and analysis of gene expression by quantitative real-time PCR (qPCR)

Details of RNA extraction, quantification, reverse-transcription (RT), application of qPCR and primer sequences used to measure the expression levels of human and baboon transcripts included in this study (pituitary hormones, sst2, sst5 and D_2_ [long (D_2_L) and total (D_2_T) isoforms] and POU1F1) have been previously reported elsewhere by our group^10^,^11^,^31^. Briefly, human and baboon frozen tissues were processed for recovery of total RNA with two commercial kits following the manufacturer’s protocols: Absolutely RNA RT-PCR Miniprep Kit (Agilent, La Jolla, CA, USA) with deoxyribonuclease treatment and AllPrep DNA/RNA/Protein Mini Kit followed by deoxyribonuclease treatment using RNase-Free DNase Set (Qiagen, Limburg, Netherlands). Total RNA from pituitary cell cultures was isolated using TRIzol Reagent (Life Technologies, Barcelona, Spain) following the manufacturer’s protocol and subsequently treated with DNase (Promega, Barcelona, Spain). Total RNA amount was quantified using the Ribogreen RNA Quantification Kit (Molecular Probes, Eugene, OR). Total RNA (1 μg for whole human and baboon tissues; ∼0.15 μg for pituitary cell cultures treated with vehicle or BIM-23A760) was reversed transcribed using random hexamers and the cDNA First Strand Synthesis kit (MRI Fermentas, Hanover, MD, USA); and the cDNAs were amplified by qPCR using a Stratagene Mx3000p real-time PCR machine and the brilliant SYBR Green QPCR Master Mix (Stratagene, La Jolla, CA, USA). Samples were run against synthetic standards (1, 10^1^, 10^2^, 10^3^, 10^4^, 10^5^, and 10^6^ copies) for each transcript of interest to estimate mRNA copy number, and a No-RT sample was used as a negative control. Thermal profile: one step at 95 °C for 10 minutes; 40 cycles of denaturation (95 °C for 30 seconds), annealing (61 °C for 1 minute), and extension (72 °C for 30 seconds); and one dissociation cycle to verify the reaction. Since it is not possible to design a specific set of primers for qPCR that only amplified the short isoform of D_2_^11^, a set of primers that amplify both, the long and short, isoforms (D_2_T) and a set of primers that only amplify the long isoform (D_2_L) were used in this study. As previously reported, to control for variations in the amount of RNA used in the RT reaction and the efficiency of the RT reaction, the expression level (copy-number) of each transcript was adjusted by ACTB (human samples^30^) or by PPIA (baboon samples^51^) expression, used as control genes based on their stability among the different samples (no significant changes were observed in the expression of these genes among experimental groups).

### Measurements of free cytosolic calcium concentration ([Ca^2+^]_i_)

As we have previously described in detail elsewhere^29^,^30^,^53^, changes in [Ca^2+^]_i_ in response to treatment with BIM-23A760 were measured in single cultured human pituitary cells (50,000 cell/well) by using fura-2AM probe (Molecular Probes, Eugene, OR, USA) and MetaFluor Software (Imaging Corp, West Chester, PA, USA). Particularly, human primary pituitary cells were plated for 36–48 h onto glass coverslips (35-mm plates); then they were incubated at 37 °C for 30 min with fura-2AM in phenol red-free DMEM containing 20 mM NaHCO_3_ (pH 7.4). Coverslips were washed with phenol red-free DMEM and placed on a Sykes-Moore chamber (Bellco Glass, Madrid, Spain) and set on an inverted microscope Eclipse TE2000-E (Nikon, Tokyo, Japan) attached to a digital camera ORCA II BT (Hamamatsu Photonics, Hamamatsu, Japan). Briefly, cells were examined for changes in [Ca^2+^]_i_ after BIM-23A760 treatment while exposed to alternating 340–380 nm light beams, and the intensity of light emission at 505 nm was measured every 5 seconds using a 40x objective with Immersion Oil Type NF (Nikon, Tokyo, Japan). Data was collected and processed using MetaFluor Software (Imaging Corp., West Chester, PA, USA). Phenol red-free DMEM and ionomycin (Sigma-Aldrich, Madrid, Spain) were used, respectively, as negative and positive controls.

### Measurements of cell viability

Cell viability was evaluated in primary cell cultures (10,000 cell/well; n = 4–5 well/treatment) treated with BIM-23A760 as compared with vehicle-treated controls using the alamar-Blue reagent (Biosource International; Camarillo, CA, USA). Specifically, cells were serum-starved for 12–16 h and then, treated with serum-free medium containing 10% alamar-Blue for 3 h, and subsequently treated for 24-, 48-, and 72-h with BIM-23A760 or vehicle-controls. Reduction of alamar-Blue was quantified exciting at 560 nm and reading at 590 nm in the FlexStation III system (Molecular Devices, Sunnyvale, CA, USA) following the manufacturer’s instructions, as previously reported^29^,^30^,^53^.

### Measurements of apoptosis

To evaluate the apoptotic rate in GHomas, 150,000 cells/well were plated and cultured for 36 h. Cell cultures were incubated for 12 h with BIM-23A760 as compared with vehicle-treated controls; after that: media were collected, centrifuged 5 min at 1,200 rpm, and the supernatant was discarded and the pellet was maintained; then cells were washed with PBS, detached with a cell scrapper and collected together with the previous pellet. Then, centrifuged for 5 min at 1,200 rpm and the supernatant was discarded while the pellet was processed following manufacturer’s instructions of Annexin-V-FITC/propidium iodide staining assay (Bender Medsystems, Barcelona, Spain) and measurement of apoptotic rate were carried out by flow cytometry (Beckman Coulter, Coulter Epics XL, Madrid, Spain).

### Statistical analysis

Statistical differences were assessed by unpaired parametric t-test (using Welch correction in the case of unequal variances) or nonparametric Mann-Whitney tests according to normality, assessed by Kolmogorov-Smirnov test. No corrections for multiple comparisons were performed. As previously reported^30^,^51^,^53^ to normalize values within each treatment and minimize intragroup variations in the different *in vitro* experiments (i.e. different age of the tissue donor, different stage of the estrus cycle or metabolic environment), the values obtained were compared with vehicle-treated controls (set at 100%). Specifically, to generate these values, individual values (adjusted by the corresponding level of control gene in the case of qPCR), within each individual experiment were divided by the mean value of the control group and multiplied times 100, and the means of these adjusted values are presented with their associated standard error. It should be emphasized that this style of data presentation does not alter the relative differences between BIM-23A760-treated and vehicle-treated groups. All data are expressed as mean ± SEM. In the cases wherein only one (n = 1) experiments could be performed due to a limitation in the availability of pituitary adenoma samples/cells, mean from tri-quadruplicate measures without error bars are depicted. p < 0.05 was considered significant; when p-values ranged between <0.1 and >0.05, a trend for significance was indicated where appropriate. All statistical analyses were performed using GraphPad Prism 6 (GraphPad Software; La Jolla, CA, USA).

## Additional Information

**How to cite this article**: Ibáñez-Costa, A. *et al*. BIM-23A760 influences key functional endpoints in pituitary adenomas and normal pituitaries: molecular mechanisms underlying the differential response in adenomas. *Sci. Rep.* **7**, 42002; doi: 10.1038/srep42002 (2017).

**Publisher's note:** Springer Nature remains neutral with regard to jurisdictional claims in published maps and institutional affiliations.

## Supplementary Material

Supplementary Table 1 and Figure 1

## Figures and Tables

**Table 1:** Demographic data of all the patients included in this study.

	n	Age, years (min-max)	Gender (male/female)
Normal pituitary	5	43 (21–69)	0/5
Somatotropinoma	22	43 (13–64)	8/14
Mixed GH-PRL adenomas	5	39 (32–60)	2/3
Prolactinoma	6	32 (18–47)	3/3
Corticotropinoma	11	38 (23–68)	1/10
Non functioning pituitary adenoma	26	58 (22–74)	14/12
Gonadotropinoma	1	35	0/1
Thyrotropinoma	3	58 (47–71)	1/2

**Table 2:** Effect of BIM-23A760 on free cytosolic calcium kinetics.

	PRC (%)	PMR (%) ± SEM	Time ± SEM
Normal pituitary-1	60.0	71.2 ± 2.4	109.4 ± 0.5
Normal pituitary-2	52.0	79.1 ± 2.2	74.2 ± 0.7
Normal pituitary-3	16.3	76.0 ± 3.0	59.1 ± 3.4
Normal pituitary-4	37.5	83.2 ± 1.1	69.7 ± 0.3
GHoma-1	52.5	65.0 ± 2.2	38.3 ± 2.4
GHoma-2	83.5	71.9 ± 6.0	42.11 ± 7.2
GHoma-3	97.5	81.3 ± 1.05	30.13 ± 0.97
GHoma-4	90.0	74.2 ± 2.8	30.0 ± 0.6
GHoma-5	80.0	68.8 ± 2.0	42.0 ± 1.7
GHoma-6	76.6	68.3 ± 8.7	47.8 ± 2.4
GHoma-7	77.3	72.5 ± 2.3	25.6 ± 2.0
GHoma-8	87.5	61.1 ± 2.2	54.3 ± 2.0
GHoma-9	65.0	74.8 ± 0.8	104.8 ± 0.2
GHoma-10	58.2	80.6 ± 3.2	38.6 ± 0.3
GHoma-11	20.0	64.8 ± 3.2	36 ± 3.5
GHoma-12	12.5	72.6 ± 4.3	61.0 ± 5.7
GHoma-13	27.5	77.4 ± 1.3	71.4 ± 4.5
GHoma-14*	90.0	243.8 ± 9.1	27.8 ± 1.6
GHoma-15*	89.5	228.4 ± 3.3	18.0 ± 7.1
GHoma-16*	86.3	254.7 ± 15.2	37.0 ± 4.4
GHoma-17*	11.3	123.3 ± 6.7	31.5 ± 3.5
GHoma-18*	97.5	199.5 ± 6.8	26.2 ± 0.7
GHoma-19*	37.5	222.7 ± 13.6	56.3 ± 3.7
GHoma-20^†^	0.0		
Mixed GH/PRLoma-1	30.0	76.9 ± 2.0	43.8 ± 2.0
Mixed GH/PRLoma-2	63.9	63.1 ± 2.4	53.0 ± 2.0
Mixed GH/PRLoma-3	32.5	77.0 ± 1.7	77.7 ± 4.5
Mixed GH/PRLoma-4	41.2	81.5 ± 2.4	26.0 ± 3.0
Mixed GH/PRLoma-5^†^	0.0		
PRLoma-1	41.0	63.0 ± 3.0	49.0 ± 2.4
PRLoma-2	25.0	59.9 ± 2.8	92.0 ± 4.5
PRLoma-3	42.9	63.0 ± 5.5	48.3 ± 4.9
PRLoma-4	9.5	88.0 ± 1.3	35 ± 0
PRLoma-5^†^	0.0		
PRLoma-6^†^	0.0		
ACTHoma-1	100.0	58.5 ± 3.8	82.5 ± 8.8
ACTHoma-2	32.5	58.5 ± 4.3	53.1 ± 3.2
ACTHoma-3	15.0	86.5 ± 3.7	48.3 ± 4.9
ACTHoma-4*	2.5	232.7 ± 0.0	20.0 ± 0.0
ACTHoma-5*	15.0	172.0 ± 17.0	28.0 ± 4.5
ACTHoma-6*	55.0	247.7 ± 13.0	67.5 ± 2.4
ACTHoma-7^†^	0.0		
ACTHoma-8^†^	0.0		
ACTHoma-9^†^	0.0		
ACTHoma-10^†^	0.0		
NFPA-1	70.7	82.4 ± 0.3	52.9 ± 24.6
NFPA-2	20.5	78.1 ± 2.3	58.4 ± 4.6
NFPA-3	42.5	81.3 ± 8.8	47.8 ± 4.8
NFPA-4	43.5	83.3 ± 0.6	65.0 ± 2.0
NFPA-5*	17.6	163.0 ± 17.0	51.0 ± 2.7
NFPA-6*	20.0	177.1 ± 18.8	30.0 ± 5.0
NFPA-7^†^	0.0		
NFPA-8^†^	0.0		
NFPA-9^†^	0.0		
NFPA-10^†^	0.0		
NFPA-11^†^	0.0		
NFPA-12^†^	0.0		
NFPA-13^†^	0.0		
NFPA-14^†^	0.0		
NFPA-15^†^	0.0		
NFPA-16^†^	0.0		
FSHoma-1	66.2	64.5 ± 0.6	52.9 ± 5.7
TSHoma-1	64.7	52.9 ± 3.0	14.0 ± 0.8
TSHoma-2^†^	0.0		

The proportion of responsive cells (PRC) showing changes in [Ca^2+^]_i_ levels in response to BIM-23A760 is indicated for all the pituitary cell culture preparations. Percentage of maximum response (PMR) and time of response to BIM-23A760 administration are also indicated. Pituitary adenomas in which an stimulatory response to BIM-23A760 was observed are indicated by an asterisk (*) while adenomas that did not respond to BIM-23A760 are indicated by a cross (^†^). The percentage of response (in %) and the time (in seconds) is indicated as mean ± SEM of 20–120 individual cells (depending on the experiment).
