# Supplementary material for: BIM-23A760 influences key functional endpoints in pituitary adenomas and normal pituitaries: molecular mechanisms underlying the differential response in adenomas
Source: Sci Rep. 2017 Feb 9;7:42002. doi: 10.1038/srep42002 (PMC5299479; doi:10.1038/srep42002)
Supplement: Supplementary Table 1 and Figure 1 [file srep42002-s1.pdf]

BIM-23A760 influences key functional endpoints in pituitary adenomas and normal pituitaries: molecular mechanisms underlying the differential response in adenomas

Alejandro Ibáñez-Costa[1,2,3,4,5], Laura M. López-Sánchez[1,3], Manuel D. Gahete[1,2,3,4,5], Esther Rivero-Cortés[1,2,3,4,5], Mari C. Vázquez-Borrego[1,2,3,4,5], María A. Gálvez[1,3,6], Andrés de la Riva[1,3,7], Eva Venegas-Moreno[8], Luis Jiménez-Reina[9], Alberto Moreno-Carazo[10], Francisco J. Tinahones[11], Silvia Maraver-Selfa[11], Miguel A. Japón[12], Juan A. García-Arnés[13], Alfonso Soto-Moreno[8], Susan M. Webb[14], Rhonda D. Kineman[15], Michael D. Culler[16], Justo P. Castaño[1,2,3,4,5]*, Raúl M. Luque[1,2,3,4,5]*.

Supplemental table-1. Absolute cDNA copy number/50ng total RNA of gene transcripts in the whole pituitary versus primary pituitary cell cultures (control groups) of female baboons, as determined by quantitative real-time PCR. Values represent means ± SEM (from 3 separate whole pituitary extracts and primary pituitary cell cultures of the same baboons).

Gene	Whole pituitary	Primary pituitary cell cultures
**sst2**	2,182 ± 229	2,018 ± 309
**sst5**	4,465 ± 357	3,809 ± 479
**D_2T**	28,269 ± 4,239	20,347 ± 2,785
**D_2L**	16,116 ± 3,077	11,420 ± 1,620
**Cyclophilin A**	79,469 ± 5,578	88,280 ± 1,626

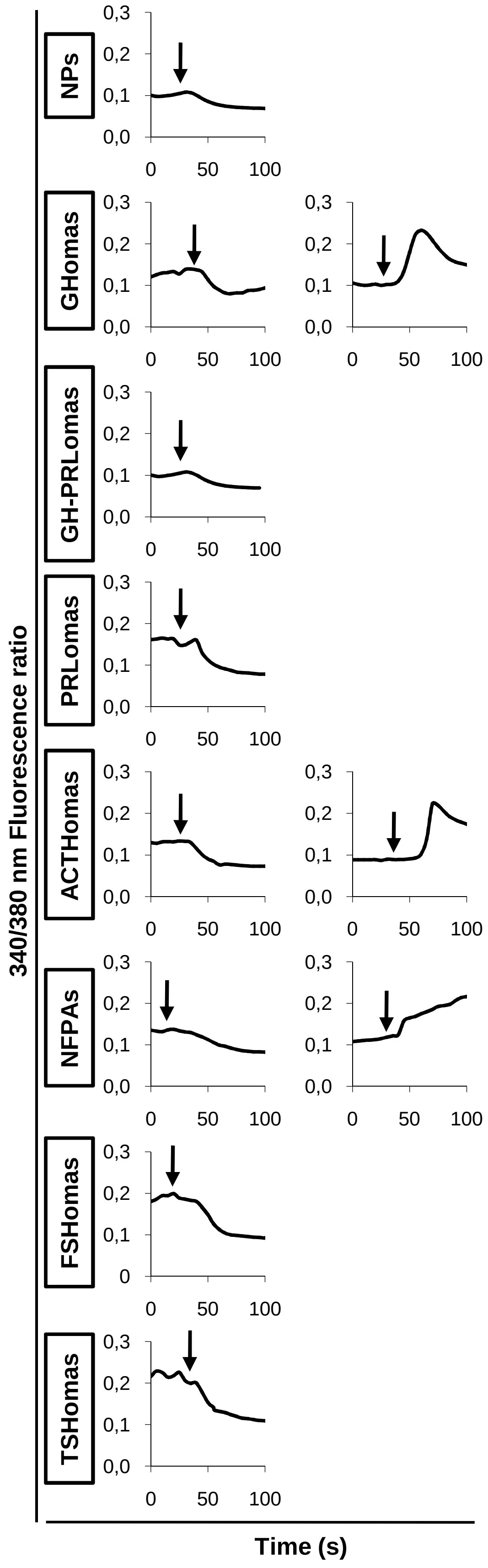

Supplemental Figure 1. Representative profiles of changes in $[Ca^{2+}]_i$ in primary cell culture from normal pituitaries (NPs, n=4) as well as from GHomas (n=20), mixed GH/PRLomas (n=5), PRLomas (n=6), ACTHomas (n=10), NFPAs (n=16), FSHomas (n=1) and TSHomas (n=2) in response to BIM-23A760 administration (100 nM; arrow).
